# A novel deletion mutation in the *TUSC3 *gene in a consanguineous Pakistani family with autosomal recessive nonsyndromic intellectual disability

**DOI:** 10.1186/1471-2350-12-56

**Published:** 2011-04-22

**Authors:** Muzammil Ahmad Khan, Muhammad Arshad Rafiq, Abdul Noor, Nadir Ali, Ghazanfar Ali, John B Vincent, Muhammad Ansar

**Affiliations:** 1 Department of Biochemistry, Quaid-i-Azam University Islamabad, Islamabad, Pakistan; 2 Molecular Neuropsychiatry & Development Lab, Neurogenetics Section, Centre for Addiction and Mental Health, Toronto, ON, Canada; 3 Center of Excellence in Biotechnology Research, King Saud University, Riyadh, Kingdom of Saudi Arabia; 4 Department of Psychiatry, University of Toronto, Toronto, Ontario, Canada

## Abstract

**Background:**

Intellectual disability (ID) is a serious disorder of the central nervous system with a prevalence of 1-3% in a general population. In the past decades, the research focus has been predominantly on X-linked ID (68 loci and 19 genes for non syndromic X linked ID) while for autosomal recessive nonsyndromic ID (NSID) only 30 loci and 6 genes have been reported to date.

**Methods:**

Genome-wide homozygosity mapping with 500 K Nsp1 array (Affymetrix), CNV analysis, PCR based breakpoint mapping and DNA sequencing was performed to explore the genetic basis of autosomal recessive nonsyndromic ID in a large Pakistani family.

**Results:**

Data analysis showed linkage at 8p23 locus with common homozygous region between SNPs rs6989820 and rs2237834, spanning a region of 12.494 Mb. The subsequent CNV analysis of the data revealed a homozygous deletion of 170.673 Kb which encompassed the *TUSC3 *gene.

**Conclusion:**

We report a novel deletion mutation in *TUSC3 *gene which is the second gene after *TRAPPC9 *in which mutation has been identified in more than one family with autosomal recessive NSID. The study will aid in exploring the molecular pathway of cognition.

## Background

Intellectual disability (ID), also frequently referred to as Mental Retardation or cognitive impairment (CI), is a condition where intelligence quotient is less than 70, there is deficiency in at least two adaptive skills like communication, reading, writing, self care etc and onset before 18 year of age [1]. Assuming a population mean IQ of 100, ID is subcategorized as mild (50-55 to 70), moderate (35-40 to 50-55), severe (20-25 to 35-40) and profound (below 20-25) [1,2].

Generally it is believed that ~25% of genetic ID patients are thought to have autosomal recessive mode of inheritance [3]. To date 30 loci including six known genes have been reported to be involved in autosomal recessive NS-ID (ARNS-ID) [4]. These include *PRSS12 *(Protease, Serine, 12 or Neurotrypsin; MIM# 606709) [5], *CRBN *(Cereblon; MIM# 609262) [6], *CC2D1A *(Coiled-coil and C2 domain containing protein 1A; MIM# 610055) [7], *GRIK2 *(Glutamate receptor, ionotropic, kainite 2; MIM#138244) [8], *TUSC3 *(Tumor suppressor candidate 3; MIM# 601385) [9,10], *TRAPPC9 *(Trafficking protein particle complex subunit 9; MIM# 611966) [11-13].

Chromosome 8 is considered an average chromosome with respect to size (146.364 Mb), number of genes (1198), repeat content and degree of segmental duplication [14]. But its p arm showed high degree of sequence variations, particularly within its distal-most ~15 megabase region. This region is believed to be of prime importance in the human genome because of the high expression pattern of nervous system related genes, and has recently been touted as a "hub" for neuropsychiatric developmental disorders [15]. Many genomic imbalances on 8p locus, such as duplication of 8p23.1-8p22.2, are associated with learning disability [16]. Also one gene for microcephaly (*MCPH1*) and one gene for NS-ID, namely *TUSC3*, have been identified on 8p.

In this study we present the clinical and molecular analysis of a consanguineous Pakistani family with autosomal recessive NS-ID, and report a novel mutation comprising deletion of the entire *TUSC3 *gene (except for the promoter and 1^st ^exon) and its down stream region at the 8p23 locus.

## Methods

### A: Sampling and DNA extraction

The family was recruited from a rural part of Sindh province of Pakistan, after getting prior approval from Institutional Review Board (Quaid-I-Azam U IRB#1- Biomedical; IORG0002926; IRB00003532), and blood samples were taken from available affected and unaffected family members and DNA was extracted from whole blood by following the standard proteinase K/phenol/chloroform isolation method. Written informed and photography consents (Translated in local Urdu language) were obtained from the parents/guardians of patients participating in this study, which conforms to Helsinki Declaration and local legislation.

### B: Clinical Assessments

Affected family members were evaluated with the help of standard questionnaire (translated and amended version of Wechsler Intelligence Scale in Urdu) for severity of disease and IQ assessments. Photographs of affected members were taken with written consent to study and publish their facial features. Two individuals from two different loops of the pedigree were selected for cranial CT scan (IV-10 and IV-14) to screen for abnormal brain anatomical features. For information on disease onset, parents were interviewed about the prenatal, perinatal and neonatal medical history of the proband.

### C: Molecular Assessments

#### (i) Genome-wide scan

Whole Genome scan was performed by using Genechip Mapping 500K array NspI chip (Affymetrix) for four affected and 2 unaffected family members and the data was analyzed using dChip and HomozygosityMapper software for homozygosity mapping and copy number analysis [17,18]. After fine mapping the region in complete family data was analyzed with Merlin for two-point and multipoint LOD score calculations. For this purpose pedigree was divided in to two loops to overcome size limitations of the Merlin.

#### (ii) PCR based deletion break-point mapping

To map the deletion breakpoints, primers were designed (for both upstream and downstream regions) between the deleted and intact SNPs identified from the microarray data, using Primer3 software (version 0.4.0) [19]. PCR was performed for both sets of primers, and only those sets (from both the distal and proximal regions) were selected that lie closest to the deleted SNPs and showed amplification. PCR was performed using a forward primer from the distal set and reverse primer from the proximal set to amplify the junction fragment. The junction fragment was then sequenced, and the exact physical co-ordinates were determined using the BLAT tool of the UCSC Genome Browser to align the sequence to the genome [20].

## Results

### Clinical description

**Figure 1:**
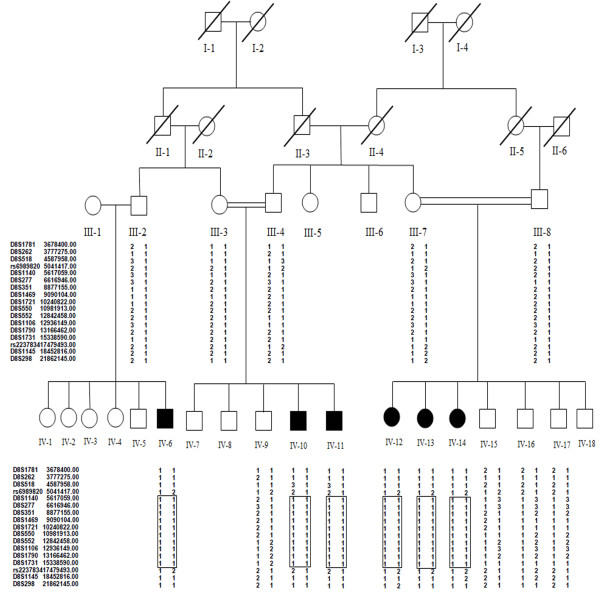
**Pedigree of a Pakistani family segregating AR-NSID**. Square represent male and circles female while black symbols represents affected individuals and clear symbols unaffected individuals. Autozygosity mapping was done for individuals IV-11, IV-12, IV-13 and IV-14. All affected individuals share a homozygous haplotype in the chromosome 8p23.1 region, which is shown in boxes. Markers with physical map distances are shown on the left side.

**Figure 2:**
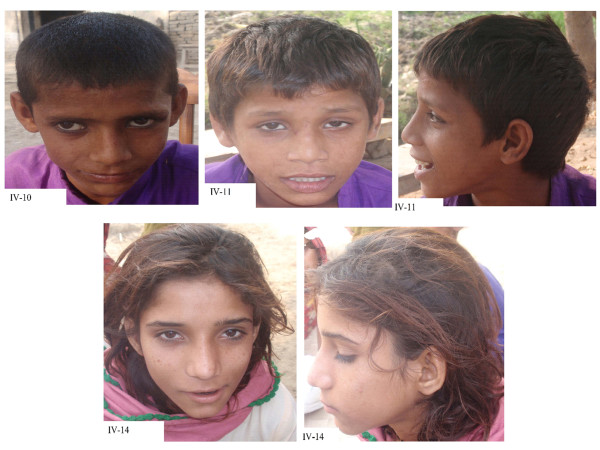
**Facial pictures of patients with front and side pose; Individual IV-10 has a minor ophthalmic issue, whereas individuals IV-11, and IV-14 reveal no apparent dysmorphism**.

The family under examination consisted of six affected individuals from three different extended branches (Figure 1) and who suffer from cognitive impairment with an IQ in the range of severe ID (25-35). Prenatal, perinatal and neonatal medical histories of all affected individuals were normal. Ophthalmological and otorhinolaryngological findings were also normal. Cranial computed tomography (CT) performed on the patients from two separate loops did not reveal any brain dysmorphology or neurologic symptoms (Data not shown). No facial dysmorphism was observed except for affected individual IV-10 who had strabismus (Figure 2). In individual IV-15 cognitive impairment was also accompanied by a form of muscular dystrophy, so this individual was not treated as affected for the purposes of the analysis which was later supported by the homozygosity and CNV data. The clinical information of the affected individuals (on the basis of questionnaire) is presented in Table 1.

**Table 1:** Summary of the clinical data of affected individuals.

Clinical Findings	IV-12	IV-14	IV-13	IV-6	IV-10	IV-11
Sex	Female	Female	Female	Male	Male	Male

Age on assessment	15 years	10 years	13 years	18 years	10 years	11 years

Developmental delay	+	+	+	+	+	+

Head Circumference	52	51 cm	51	Not Available	52 cm	50 cm

Speech Development	+	+	+	+	+	+

Dysmorphic feature	-	-	-	-	-	-

Skeletal Problem	-	-	-	-	-	-

Ophthalmological problem	-	-	-	-	-	-

Epilepsy	-	+	-	-	-	-

Mental retardation	Severe	Severe	Severe	Severe	Severe	Severe

Growth	Normal	Normal	Weak	Normal	Normal	Normal

Schooling	-	-	-	-	-	-

Learning Disability	+	+	+	+	+	+

Muscular dystrophy	-	-	-	-	-	-

Self biting	-	-	-	-	-	-

Each column indicates the data of an individual and row presents the category of clinical symptoms, (- indicates absence, + indicates presence).

### Molecular studies

#### (i) Genome-wide Homozygosity mapping and CNV analysis

**Figure 3:**
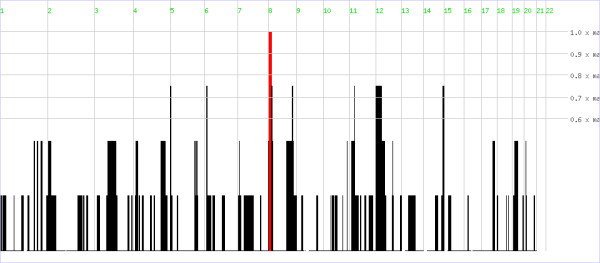
**Graphical representation of the homozygous by decent (HBD) regions identified by homozygosity mapper**. The red bar indicates the HBD identified on chromosome 8.

Genome wide homozygosity mapping revealed homozygosity-by-descent (HBD or autozygosity) among four affected individuals (IV-11, IV-12, IV-13) on 8p23 (Figure 3) between SNPs rs6989820 and rs2237834, which delineates a critical region of 12.494 Mb {UCSC genome Browser, May 2004 (NCBI35/hg17)}. In order to confirm segregation of the identified HBD region in the entire family STS markers D8S1781, D8S262, D8S518, D8S1140, D8S277, D8S351, D8S1469, D8S1721, D8S550, D8S552, D8S1106, D8S1790, D8S1731, D8S1145 and D8S298 were genotyped. Haplotypes were generated and are presented in Figure 1, which also indicate the segregation of a minimum critical region flanked by above mentioned SNPs. Linkage analysis yielded two-point and multipoint LOD scores above 3 and 5 respectively at several markers (Table 2). This region contained a total of 96 known genes {UCSC Genome Browser, May 2004 (NCBI35/hg17)} including *MCPH1 *and *TUSC3*, which have already been reported to be involved in microcephaly with ID. Initially *MCPH1 *gene was sequenced to detect pathogenic mutation in this family but the sequence analysis only revealed the presence of known SNPs in exon 1 (rs2305023), 6 (rs2442513) and 8 (rs930557 and rs2920676). Subsequent CNV analysis of microarray data showed homozygous deletion of 170.673 Kb region in all affected individuals. The deletion encompassed almost the entire *TUSC3 *gene (minus the promoter and first exon) and its downstream region.

**Table 2:** Two point and multipoint LOD score between identified HBD and chromosome 8 markers.

Markers	Genetic Position in cM (Rutgers map, build 36)	Physical Position in bp {Feb.2009 (GRCh37/hg19}	Two point LOD Score	Multipoint LOD Score
D8S1781	6.8	3678400	2.2342	4.2286

D8S262	7.13	3777275	1.0814	4.2143

D8S518	9.88	4587958	0.5706	1.456

rs6989820	-	5041417	-3.8284	-4.0196

D8S1140	11.96	5617059	3.1114	2.9368

D8S277	14.92	6616946	2.9565	5.0000

D8S351	18.94	8877155	3.0286	5.1617

D8S1469	19.38	9090104	1.523	5.1693

D8S1721	19.7	10240822	1.8241	5.1717

D8S550	20.85	10981913	- Infinity	- Infinity

D8S552	24.76	12842458	2.9823	5.1646

D8S1106	24.76	12936149	3.2113	5.1646

D8S1790	26.35	13166462	3.2843	5.1229

D8S1731	27.95	15338590	1.3825	4.5748

rs2237834	-	17479493	-3.8284	-4.4686

D8S1145	32.78	18452816	- Infinity	- Infinity

D8S298	40.11	21862145	- Infinity	- Infinity

#### (ii) Deletion breakpoint mapping

**Figure 4:**
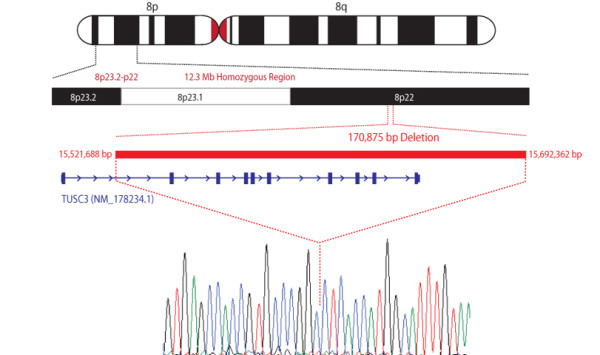
**Ideogram depicting the 12.3 Mb homozygous region on 8p23 locus**. Red bar is representing total region of deletion which contains *TUSC3 *gene shown in blue color bar. The dotted line from red bar over the sequence chromatogram indicates the junction point.

The CNV analysis of microarray data revealed that in all affected individuals no hybridization signals were generated for 22 SNPs on chromosome 8; from rs4258002 to rs352769 as borderline deleted SNPs. With the evidence of microarray data of four affected individuals, size of deletion fragment was mapped by PCR amplification and sequencing of junction fragment by designing primer (forward primer 5 '-TGCTCTCTGCTCTTCCTCGT-3'; reverse primer: 5'-CTTTCCTGGCAAGCTGCTAC-3') between deleted and intact SNPs (between rs10094375 and rs4258002 towards the distal end, and rs352769 and rs6530906 proximally). The BLAT search for the junction fragment sequence against human genome revealed the actual physical co-ordinates of deletion as being between 15521688 bp to 15692362 bp, and spanning a region of 170.673 Kb. The deletion was also checked in all available family members by using *TUSC3 *exon 3 and 5 and junction fragment primers. Both of these primer sets showed amplification in the normal individuals which represented the heterozygous segregation of this deletion except in individual IV-15. All the affected individuals of this family were homozygous for the deletion, and no homozygous or heterozygous deletion carriers were identified among 276 unrelated healthy Pakistani individuals. This deletion encompassed almost the entire *TUSC3 *gene (minus the promoter and first exon) and its downstream region (Figure 4). According to the database of genomics variants [21] 57 CNVs have been reported in HBD region identified in this family, but 27 involve *TUSC3 *gene. The detailed analysis of the CNV data indicates that all the presently identified *TUSC3 *involving CNVs exist in heterozygous state.

### Discussion and Conclusion

Autosomal recessive NSID accounts for ~25% of genetic ID cases and may be more common than X linked cases, however, the molecular basis of AR-NSID is relatively poorly known because of clinical and genetic heterogeneity, and the absence of distinguishing clinical criteria. In non-syndromic ID, cognitive deficit is the sole clinical feature among the patients. It is suggested that ID may be caused by the disruption of the biological and molecular processes in the nervous system such as neuronal differentiation and synaptic plasticity, synaptic vesicles cycling and gene expression, regulation profiling etc [22]. There are number of co-translational and post-translational modifications required for protein stability and proper protein folding into its 3D structure, which are essential for normal protein function. One such post-translational modification, N-glycosylation, has previously reported to be involved in non-syndromic X-linked ID at a *TUSC3 *paralogous gene, *IAP *[9].

In the current study we analyzed a four generation Pakistani family with 6 affected individuals having severe psychomotor developmental delay, segregating autosomal recessive mode of inheritance. The major clinical presentations of all patients were normal except for the cognitive dysfunction. However comparison of the clinical data (Biometric and neurological data) of our family with the patients of earlier reports did not reveal any significant difference in the phenotypic expression [9,10]. *TUSC3 *is the 5^th ^gene in autosomal recessive NSID and the 2^nd ^in which a 3^rd ^mutation has been identified, after *TRAPPC9*. Our study will aid in diagnostic assessment of AR-NSID individuals.

## Competing interests

The authors declare that they have no competing interests.

## Authors' contributions

MAK did the DNA extraction, microarray data analysis, PCR based breakpoint mapping and sequencing of junction fragments, MAR and AN performed microarray data analysis, NA and GA recruited, sampled and done clinic work up while the project designing and funding was arranged by MA and JBV. All authors have read and approved the final manuscript.

## Pre-publication history

The pre-publication history for this paper can be accessed here:

http://www.biomedcentral.com/1471-2350/12/56/prepub
